# Prostate cancer epidemiology and prognostic factors in the United States

**DOI:** 10.3389/fonc.2023.1142976

**Published:** 2023-10-12

**Authors:** Saimaitikari Abudoubari, Ke Bu, Yujie Mei, Abudukeyoumu Maimaitiyiming, Hengqing An, Ning Tao

**Affiliations:** ^1^ Department of Radiology, The First People’s Hospital of Kashi Prefecture, Kashi, Xinjiang, China; ^2^ College of Public Health, Xinjiang Medical University, Urumqi, Xinjiang, China; ^3^ The First Affiliated Hospital, Xinjiang Medical University, Urumqi, Xinjiang, China; ^4^ Xinjiang Clinical Research Center for Genitouriary System, Urumqi, Xinjiang, China

**Keywords:** prostate cancer, epidemiologic trends, specific survival, predictive models, nomogram

## Abstract

**Objective:**

Using the latest cohort study of prostate cancer patients, explore the epidemiological trend and prognostic factors, and develop a new nomogram to predict the specific survival rate of prostate cancer patients.

**Methods:**

Patients with prostate cancer diagnosed from January 1, 1975 to December 31, 2019 in the Surveillance, Epidemiology, and End Results Program (SEER) database were extracted by SEER stat software for epidemiological trend analysis. General clinical information and follow-up data were also collected from 105 135 patients with pathologically diagnosed prostate cancer from January 1, 2010 to December 1, 2019. The factors affecting patient-specific survival were analyzed by Cox regression, and the factors with the greatest influence on specific survival were selected by stepwise regression method, and nomogram was constructed. The model was evaluated by calibration plots, ROC curves, Decision Curve Analysis and C-index.

**Results:**

There was no significant change in the age-adjusted incidence of prostate cancer from 1975 to 2019, with an average annual percentage change (AAPC) of 0.45 (95% CI:-0.87~1.80). Among the tumor grade, the most significant increase in the incidence of G2 prostate cancer was observed, with an AAPC of 2.99 (95% CI:1.47~4.54); the most significant decrease in the incidence of G4 prostate cancer was observed, with an AAPC of -10.39 (95% CI:-13.86~-6.77). Among the different tumor stages, the most significant reduction in the incidence of localized prostate cancer was observed with an AAPC of -1.83 (95% CI:-2.76~-0.90). Among different races, the incidence of prostate cancer was significantly reduced in American Indian or Alaska Native and Asian or Pacific Islander, with an AAPC of -3.40 (95% CI:-3.97~-2.82) and -2.74 (95% CI:-4.14~-1.32), respectively. Among the different age groups, the incidence rate was significantly increased in 15-54 and 55-64 age groups with AAPC of 4.03 (95% CI:2.73~5.34) and 2.50 (95% CI:0.96~4.05), respectively, and significantly decreased in ≥85 age group with AAPC of -2.50 (95% CI:-3.43~-1.57). In addition, age, tumor stage, race, PSA and gleason score were found to be independent risk factors affecting prostate cancer patient-specific survival. Age, tumor stage, PSA and gleason score were most strongly associated with prostate cancer patient-specific survival by stepwise regression screening, and nomogram prediction model was constructed using these factors. The Concordance indexes are 0.845 (95% CI:0.818~0.872) and 0.835 (95% CI:0.798~0.872) for the training and validation sets, respectively, and the area under the ROC curves (AUC) at 3, 6, and 9 years was 0.7 or more for both the training and validation set samples. The calibration plots indicated a good agreement between the predicted and actual values of the model.

**Conclusions:**

Although there was no significant change in the overall incidence of prostate cancer in this study, significant changes occurred in the incidence of prostate cancer with different characteristics. In addition, the nomogram prediction model of prostate cancer-specific survival rate constructed based on four factors has a high reference value, which helps physicians to correctly assess the patient-specific survival rate and provides a reference basis for patient diagnosis and prognosis evaluation.

## 1 Introduction

Prostate cancer (PCa) is one of the leading causes of cancer-related deaths (1) and currently the second most common male malignancy worldwide (2). 375 304 deaths from prostate cancer were reported worldwide in 2020 (3). The incidence and mortality rates of prostate cancer vary greatly from country to country, and even within a single country, the incidence and mortality rates of prostate cancer vary greatly in different regions (4). Studies have reported the highest incidence of prostate cancer in Western and Northern Europe, North America and Australia/New Zealand, with intermediate incidence in Eastern Europe, South America, South Africa and Western Asia, and the lowest incidence in South and East Asia and other parts of Africa. Southern Africa, the Caribbean, and South America had the highest mortality rates. Europe, North and Central America, and Australia/New Zealand have intermediate mortality rates, and Asia had the lowest mortality rates (5).

In addition, the increasing number of articles published each year on prostate cancer is evidence that the global interest in prostate cancer has been increasing. Although the incidence and prevalence of prostate cancer are thought to have increased over the last few decades, there is a lack of recent data on the epidemiological characteristics and survival analysis of prostate cancer patients. On the other hand, most studies on prostate cancer are based on a small number of cases in a single institution and lack reliability. Therefore, in this study, we conducted a population-based study using information from the Surveillance, Epidemiology, and End Results (SEER) of the American Institute for Cancer Research to systematically analyze the epidemiologic, clinical, and prognostic characteristics of prostate cancer.

The prognosis of prostate cancer patients remains difficult to assess, although there is an increasing focus on the prognosis and survival of prostate cancer patients. The current prognostic analysis of prostate cancer is still mainly based on the American Joint Committee on Cancer tumor TNM staging system (6). This system assesses the prognosis of patients based on tumor volume (T), regional lymph node tumor invasion (N), and distant metastases (M). However, the TNM staging system is not yet able to adequately assess patient-specific survival, and more reliable predictive evaluation indicators need to be explored (7). Among the currently available predictive tools, nomogram is considered to be the most accurate and characteristic method for predicting prognosis of cancer patients (8). To our knowledge, few studies have used nomogram to predict the prognosis of prostate cancer patients. In this study, a more detailed nomogram was developed based on a relatively large cohort of prostate cancer patients in the SEER database to predict the 3, 6, and 9 year specific survival rates of prostate cancer patients to provide a reference for patient treatment and prognostic evaluation.

## 2 Materials and methods

### 2.1 data sources

The SEER database used for this study is an authoritative source of information on cancer epidemiology (incidence and prevalence) and clinical characteristics (primary tumor site, tumor morphologic features and stage of diagnosis, first course of treatment, and life-state follow-up) in the United States. Patients aged 15 years and older with prostate cancer diagnosed from January 1, 1975 to December 31, 2019 were obtained through SEER*Stat 8.4.1 software and analyzed for epidemiological trends in prostate cancer. General clinical data and follow-up data of 105 135 patients with prostate cancer diagnosed by pathology from January 1, 2010 to December 1, 2019 were also collected for analysis of prognostic influencing factors. Inclusion criteria: (1) patients with prostate cancer clearly diagnosed by pathology; (2) complete general clinical and follow-up data; (3) age ≥ 15 years. Exclusion criteria: (1) those with unclear pathological findings; (2) those with unclear general clinical information, etc. The data used in this study were freely available and publicly available. Therefore, review and informed consent were exempted.

### 2.2 Collection of clinical data related to prognostic analysis

Clinical data with serious missing wsa excluded from the database, and finally age, race, PSA, bone metastases, lung metastases, tumor grade, tumor stage, gleason score, and follow-up-related information were included. Follow-up-related information included specific survival time and follow-up outcome. The specific survival time was defined as the time interval from the date of diagnosis to the date of death due to tumor recurrence of the patient, and the follow-up outcomes included death from tumor-related causes during follow-up or the end of follow-up (survival or death from other causes). All of the above information is described in detail in the SEER database.

### 2.3 Tumor stage, tumor grade and race of study subjects

We used the SEER staging system in our study. Tumor stage was divided into different metastatic conditions such as localized, regional and distant metastasis. Localized prostate cancer was defined as a tumor that was completely confined to the organ of origin. Regional prostate cancer was defined as beyond the boundaries of the organ of origin, directly into surrounding organs or tissues, through the lymphatic system into regional lymph nodes, or through a combination of extension and regional lymph nodes. Finally, distant metastasis was defined as the appearance of metastatic lesions in organs or tissues relatively distant from the site of the primary cancer. Since tumor stage-related data in the SEER database was only recorded from 1998 to 2017, only data between 1998 and 2017 was analyzed for tumor stage-related data. For the tumor grade, the SEER classification scheme systematically classified cases into 4 classes: G1: highly differentiated; G2: moderately differentiated; G3: poorly differentiated; and G4: undifferentiated. Patients were classified into the following 4 racial categories: white people, black people, Asian and Pacific Islander, American Indian and Alaska Native.

### 2.4 Statistical analysis

SEER*Stat 8.4.1 software was used to calculate age-adjusted incidence, limited persistence prevalence (10 and 20 year prevalence), and mortality from 1975 to 2019. The Joinpoint 4.9.1 software was used to characterize incidence trends by combining annual percentage change (APC) and average annual percentage change (AAPC) calculated by point regression. The logarithm of the age-adjusted rates for each year were first regressed over time and then the annual percentage change were calculated using a slope transformation. APC and AAPC were comparable at different scales, allowing comparison of other incidence rates between malignancy cohorts. The entire sample set collected from January 1, 2010 to December 1, 2019 was also randomly divided 2:1 into training and validation sets (random number seed = 105 135), training set (n = 70 090), and validation set (n = 35 045). SPSS 25.0 software was used to statistically analyze the collected data, and the count data was described using percentages (%). One-way Cox regression was used to analyze the influential factors associated with prostate cancer-specific survival. Factors that were statistically significant in the one-way Cox regression analysis were included in the multi-factor Cox regression to analyze the independent risk factors associated with prostate cancer-specific survival. Eviews 12.0 software was used to calculate the Akaike Information Criterion value (AIC) of each independent risk factor, and a larger AIC indicated that the factor was more important to the model, and all independent risk factors were ranked according to the AIC value, and the factors were gradually included in the model according to the ranking, while R4.1.2 software (car, rms, pROC, timeROC, ggDCA, survival packages), The larger the C index is, the more accurate the model prediction is, and evaluate whether the newly added factors make the C index of the model improve. The reliability of the model was assessed by plotting the ROC curve and calculating the AUC. Calibration curves (using 1000 bootstrap auto-sampling method) were plotted to validate the model. The test level was 0.05.

## 3 Results

### 3.1 Patient characteristics

From the SEER database, a total of 1 366 129 prostate cancer patients (mean age at diagnosis, 67.90 ± 9.31 years; median age 68.00 (61.00,75.00) years were identified from 1975 to 2015. Among these, 264 450 (19.36%) were under 60, 511 638 (37.45%) were 60 to 69, 424 216 (31.05%) were 70 to 79, and 165 825 (12.14%) were 80 years and above. 1 091 443 (79.89%) were white people, 173 154 (12.67%) were black people, 63 014 (4.61%) were Asian and Pacific Islander patients, 4 896 (0.36%) were American Indian and Alaska Native patients, and 33 622 (2.46%) were patients of unknown race. In addition, of the 1 145 591 (83.86%) prostate cancers with known tumor grade, 125 356 (9.18%) were G1, 593 294 (43.43%) were G2, 421 653 (30.86%) were G3, and 5 288 (0.39%) were G4. Of the 1 041 770 (76.25%) prostate cancers with known tumor stage, 845 925 (61.92%) were Localized, 134 484 (9.84%) were regional, and 61 361 (4.49%) were distant metastases (**Table 1**).

**Table 1:** Baseline characteristics of prostate cancer patients in SEER database.

Characteristic	1975-1991 (n)	1992-1999 (n)	2000-2019 (n)	Overall [n(%)]
	128 873	173 826	1 063 430	1 366 129(100.00%)
Age(Y)				
<60	9 450	23 811	231 189	264 450(19.36%)
60~69	38 786	59 082	413 770	511 638(37.45%)
70~79	52 588	65 957	305 671	424 216(31.05%)
≥80	28 049	24 976	112 800	165 825(12.14%)
Tumor grade				
G1	33 770	20 416	71 170	125 356(9.18%)
G2	44 085	102 205	447 004	593 294(43.43%)
G3	25 421	34 900	361 332	421 653(30.86%)
G4	2 113	983	2 192	5 288(0.39%)
Unknown	23 484	15 322	181 732	220 538(16.14%)
Race				
White people	115 139	143 822	832 482	1 091 443(79.89%)
Black people	8 214	17 237	147 703	173 154(12.67%)
AI/AN	–	703	4 193	4 896(0.36%)
Asian/P Islander	–	10 427	52 587	63 014(4.61%)
Unknown	5 520	1 637	26 465	33 622(2.46%)
Tumor stage				
Localized	–	32 649	813 276	845 925(61.92%)
Regional	–	6 332	128 152	134 484(9.84%)
Distant	–	2 259	59 102	61 361(4.49%)
Unstaged	128 873	132 586	62 900	324 359(23.74%)

### 3.2 Annual incidence rate

**Figure 1:**
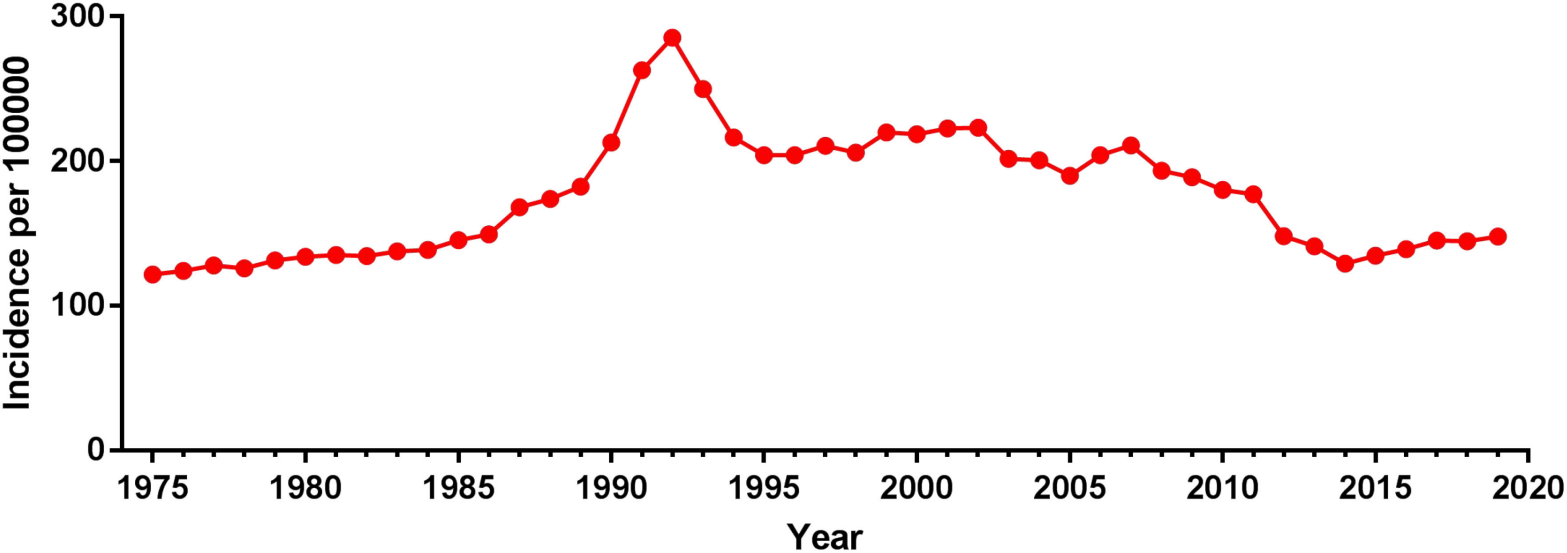
Incidence of prostate cancer over time.

**Figure 2:**
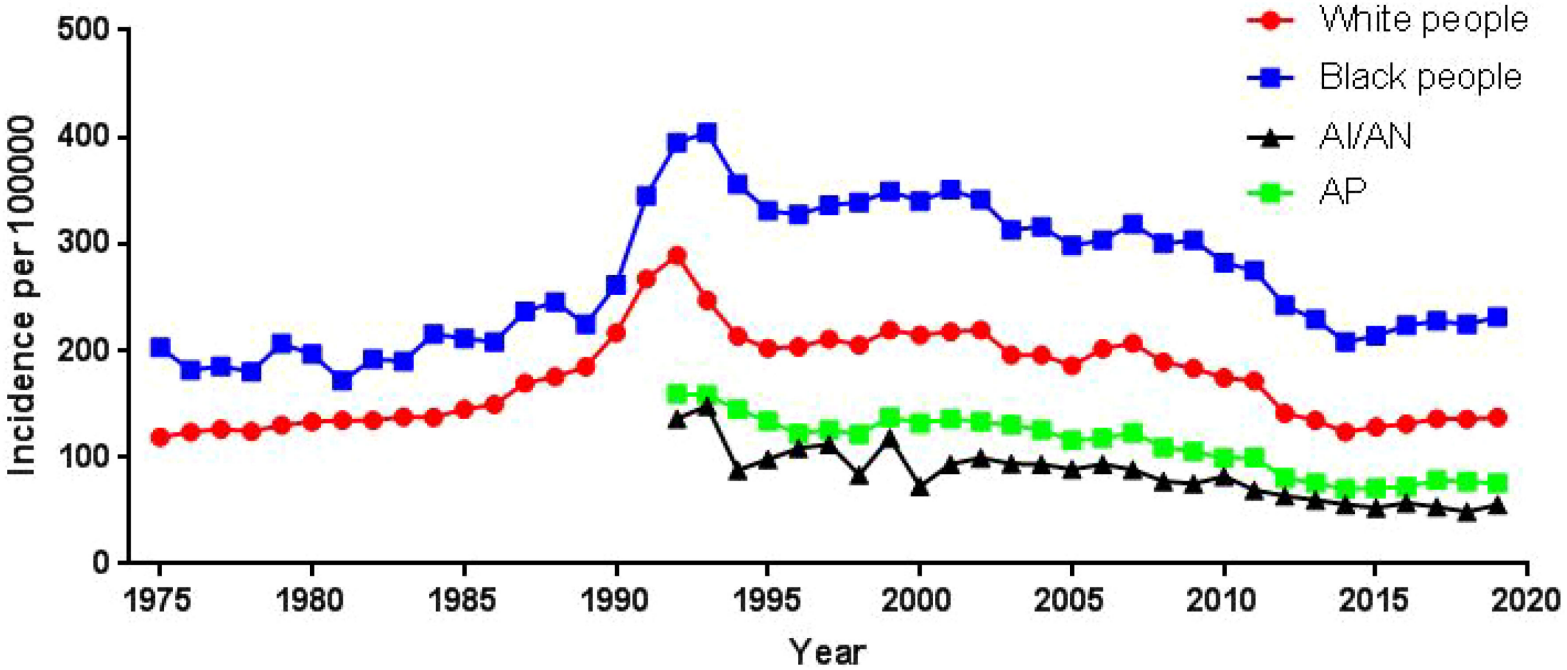
Incidence of prostate cancer over time by race.

**Figure 3:**
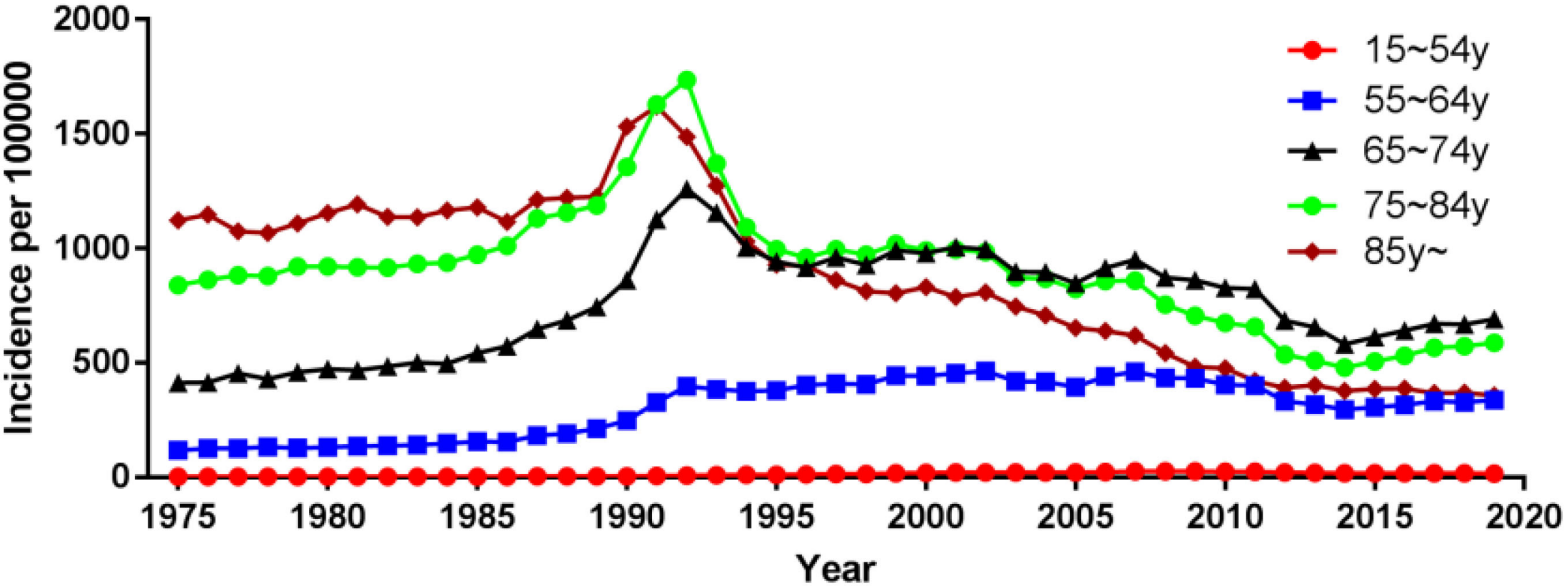
Incidence of prostate cancer over time by age.

Using population data from the SEER database, we calculated the annual age-adjusted incidence rate of prostate cancer per 100 000 persons with reference to the standard 2000 US population. The age-adjusted incidence rate of prostate cancer was 121.65 cases per 100 000 persons in 1975 and 147.86 cases per 100 000 persons in 2019, with an AAPC (95% CI) of 0.45 (- 0.87~1.80), and detailed incidence data were presented in **Table 2**; **Figure 1**, and **Supplementary Table 1**. Using data from the SEER database, long-term trends in prostate cancer incidence among different races can be explored. Incidence rates for different races did not change significantly between 1975 and 2019, with AAPC (95% CI) of 0.31 (-1.01~1.65), 0.61 (-1.10~2.35) and 0.65 (-0.51~1.83) for white people, black people, and other races, respectively. Since more detailed information on race was recorded in SEER 12 (1992-2019), we could explore the incidence trends of these races in further detail. Among White people, Black people, American Indians and Alaska Natives, Asian and Pacific Islanders, the incidence of prostate cancer decreased between 1992 and 2019, with AAPC(95% CI) of -2.65 (-4.17~-1.10), -2.09 (-3.36~-0.81), -3.40 (-3.97~-2.82), and -2.74 (-4.14~-1.32) (**Figure 2**; **Supplementary Table 1**). Among the different ages, the incidence rate increased significantly in 15-54 and 55-64 age groups, with AAPC(95% CI) of 4.03 (2.73~5.34) and 2.50 (0.96~4.05), respectively; the incidence rate decreased significantly in ≥85 age group, with AAPC(95% CI) of -2.50 (-3.43~-1.57); in 65- 74 and 75-84 age group remained unchanged, with AAPC(95% CI) of 1.21 (-0.11~2.54) and -0.79 (-1.95~0.38), respectively (**Figure 3**; **Supplementary Table 1**).

**Table 2:** Incidence of prostate cancer over time.

Registry	Year	Rate (per 100 000 persons)	Lower CI	Upper CI	Number of PC cases (n)	Number at risk (n)
SEER8	1975	121.65	118.03	125.35	4 771	5 940 293
	1976	124.24	120.62	127.93	4 997	6 066 708
	1977	127.86	124.26	131.54	5 305	6 183 531
	1978	125.83	122.29	129.43	5 349	6 304 757
	1979	131.26	127.69	134.90	5 691	6 434 477
	1980	133.75	130.19	137.38	5 949	6 562 981
	1981	135.13	131.58	138.74	6 123	6 661 878
	1982	134.33	130.85	137.87	6 282	6 741 691
	1983	137.68	134.21	141.22	6 596	6 832 059
	1984	138.57	135.13	142.08	6 777	6 929 434
	1985	145.33	141.85	148.87	7 310	7 027 803
	1986	149.27	145.81	152.80	7 711	7 129 499
	1987	168.00	164.38	171.67	8 902	7 216 079
	1988	173.84	170.21	177.54	9 417	7 308 895
	1989	182.31	178.65	186.03	10 168	7 387 431
	1990	212.91	208.99	216.88	12 071	7 488 078
	1991	262.79	258.53	267.09	15 454	7 602 113
SEER12	1992	285.32	281.73	288.94	25 512	12 087 125
	1993	249.75	246.46	253.08	23 029	12 209 948
	1994	216.48	213.46	219.53	20 453	12 308 371
	1995	204.18	201.28	207.11	19 680	12 433 844
	1996	204.01	201.15	206.90	20 053	12 577 090
	1997	210.64	207.77	213.55	21 096	12 779 857
	1998	205.83	203.02	208.67	21 039	12 984 930
	1999	219.84	216.97	222.74	22 964	13 166 050
SEER17	2000	218.53	216.62	220.45	51 294	28 494 316
	2001	222.51	220.60	224.43	53 328	28 912 565
	2002	223.11	221.22	225.00	54 904	29 275 401
	2003	201.61	199.84	203.40	50 863	29 590 069
	2004	200.65	198.90	202.42	51 824	29 966 562
	2005	189.98	188.30	191.68	50 252	30 276 193
	2006	204.24	202.51	205.97	55 561	30 614 378
	2007	210.93	209.20	212.67	59 087	30 968 322
	2008	193.46	191.83	195.10	56 135	31 353 874
	2009	188.99	187.41	190.58	56 767	31 735 085
	2010	180.10	178.57	181.63	55 618	32 080 579
	2011	177.21	175.72	178.71	56 408	32 406 115
	2012	148.02	146.68	149.36	48 781	32 731 169
	2013	141.04	139.75	142.33	47 902	33 029 532
	2014	129.14	127.92	130.36	45 135	33 343 112
	2015	134.64	133.42	135.87	48 430	33 670 052
	2016	139.17	137.95	140.41	51 436	33 969 818
	2017	145.23	143.99	146.47	55 049	34 223 179
	2018	144.73	143.51	145.96	56 010	34 428 001
	2019	147.86	146.64	149.08	58 646	34 599 429

The incidence of prostate cancer among white people increased in the 15-54 and 55-64 age groups, with AAPC (95% CI) of 3.61 (1.92~5.32) and 2.24 (0.96~3.53), respectively; decreased in and ≥85 age group, with AAPC (95% CI) of -2.54 (-3.56~-1.50); and remained unchanged in the 65-74 and 75- 84 age groups remained unchanged, with AAPC (95% CI) of 1.08 (-0.22~2.40) and -0.89 (-2.06~0.29), respectively (**Supplementary Figure 1**; **Supplementary Table 1**). The incidence of prostate cancer among black people increased in the 15-54 age group with an AAPC (95% CI) of 4.73 (2.20~7.31); decreased in the ≥85 age group with an AAPC (95% CI) of -2.46 (-3.30~-1.62); and remained unchanged in the 55-64, 65-74, and 75-84 age groups with an AAPC (95% CI) of 1.97 (-0.76~4.78), 1.00 (-0.84~2.87), and -0.97 (-3.19~1.31), respectively (**Supplementary Figure 2**; **Supplementary Table 1**). The incidence of prostate cancer among American Indians and Alaska Natives remained unchanged in the 15-54 age group with an AAPC (95% CI) of -0.87 (-2.45~0.74); it decreased in the 55-64, 65-74, 75-84 and ≥85 age groups with an AAPC (95% CI) of -2.92 (-3.83~-2.00), -3.31 (-4.03~-2.59), -3.57 (-4.53~-2.61), and -5.05 (-6.77~-3.29), respectively (**Supplementary Figure 3**; **Supplementary Table 1**). The incidence of prostate cancer among Asian and Pacific Islanders increased in the 15-54 age group with an AAPC (95% CI) of 1.61 (0.04~3.21); remained unchanged in the 55-64 age group with an AAPC (95% CI) of 0.66 (-0.71~2.05); decreased in the 65-74, 75-84 and ≥80 age groups with an AAPC (95% CI) of -1.99 (-3.34~-0.62), -4.05 (-6.08~-1.98), and -6.03 (-6.58~-5.49), respectively (**Supplementary Figure 4**; **Supplementary Table 1**).

### 3.3 Incidence and prevalence of prostate cancer by tumor stage and tumor grade

**Figure 4:**
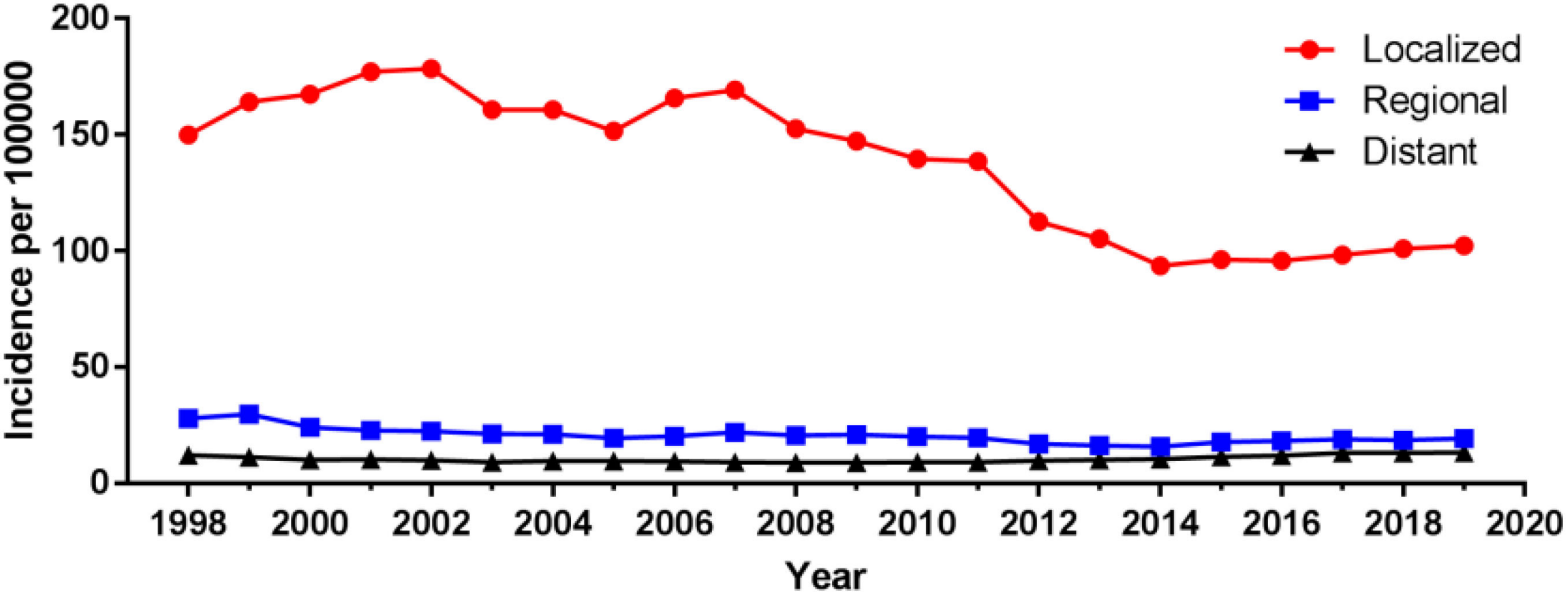
Incidence of prostate cancer over time by tumor stage.

**Figure 5:**
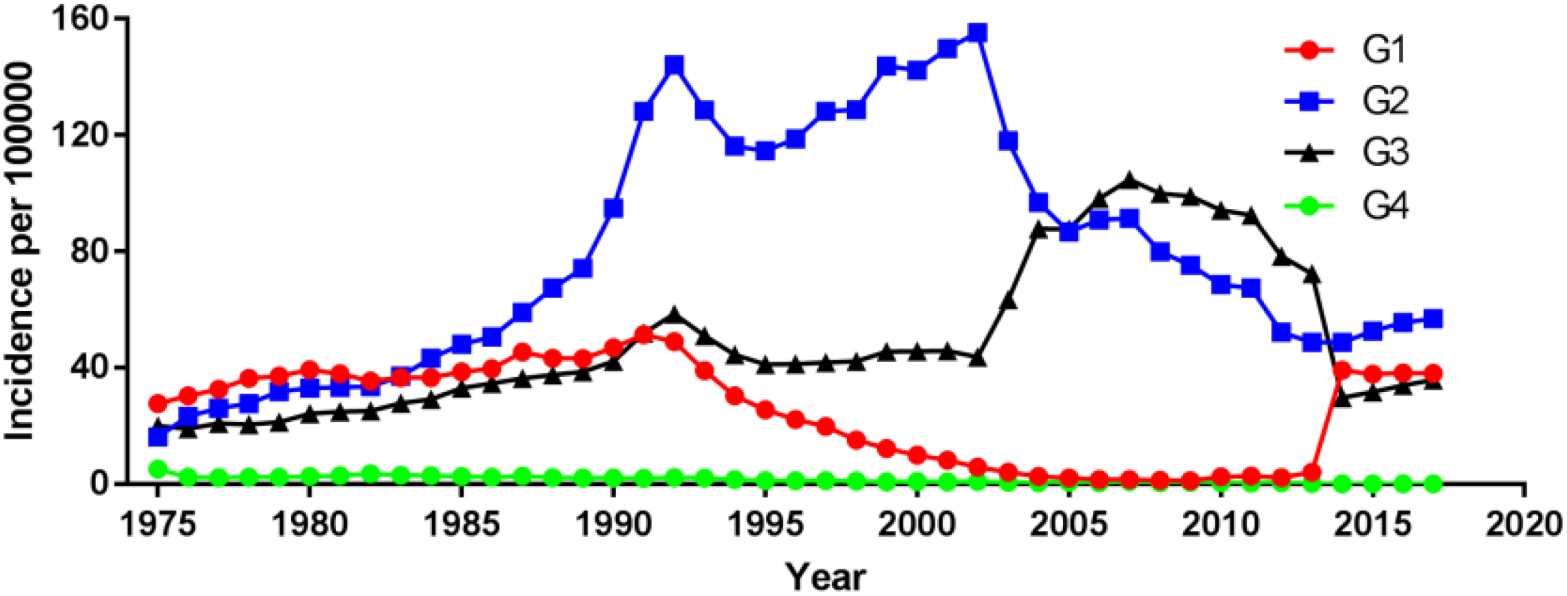
Incidence of prostate cancer over time by tumor grade.

Among different tumor stages, the incidence of localized prostate cancer decreased from 149.90 cases per 100 000 in 1998 to 102.29 cases per 100 000 in 2019, with an AAPC (95% CI) of -1.83 (-2.76~-0.90). The incidence of regional and distant metastatic prostate cancer remained unchanged, with an AAPC (95% CI) of -1.77 (-3.91~0.43) and 0.57 (-0.80~1.96), respectively (**Figure 4**; **Supplementary Table 1**). For different tumor grade, the incidence of G2 prostate cancer increased the most, from 16.20 cases per 100 000 in 1975 to 56.93 cases per 100 000 in 2017, with an AAPC (95% CI) of 2.99 (1.47~4.54), followed by G3 prostate cancer with an AAPC (95% CI) of 1.77 (0.08~3.48). The incidence of G4 prostate cancer decreased, with an AAPC (95% CI) of -10.39 (-13.86~-6.77). The incidence of G1 prostate cancer remained unchanged, with an AAPC (95% CI) of 0.47 (-1.95~2.95); (**Figure 5**; **Supplementary Table 1**).

In addition, the 20-year limited-duration prevalence of prostate cancer increased significantly from 0.20918% in 2000 to 1.87472% in 2019 (**Supplementary Figure 5**). Detailed 20-year and 10-year limited-duration prevalence and absolute counts are presented in **Table 3**. Among prostate cancers with different tumor stage, the greatest increase in prevalence was seen for localized prostate cancer (from 0.07393% in 1998 to 1.5752% in 2017), followed by regional prostate cancer (from 0.01404% in 1998 to 0.27791% in 2017) (**Supplementary Figure 6**). For the different tumor grade, the greatest increase in the prevalence was observed in G2 prostate cancer (from 0.12456% in 1998 to 1.01298% in 2017) (**Supplementary Figure 7**).

**Table 3:** 10-year and 20-year prevalence of prostate cancer.

Year	20-year duration Prevalence (%)	20-year Count (n)	10-year duration Prevalence (%)	10-year Count (n)
2000	0.20918	22079		
2001	0.41265	51308		
2002	0.60290	101463		
2003	0.77411	148580		
2004	0.93140	191490		
2005	1.07618	233094		
2006	1.21475	274007		
2007	1.35672	320617		
2008	1.47347	362921		
2009	1.57995	403756		
2010	1.66610	441745	0.17401	53964
2011	1.74986	480462	0.34321	108665
2012	1.79398	512183	0.47873	155697
2013	1.82431	538811	0.59424	198064
2014	1.83763	560611	0.68965	235364
2015	1.84864	582321	0.77976	272445
2016	1.86587	606070	0.87225	311511
2017	1.88303	632081	0.96536	352969
2018	1.89545	657413	1.05328	393784
2019	1.91003	683806	1.13858	435122

### 3.4 Trends in age at diagnosis

We calculated the mean age at diagnosis for prostate cancer patients by tumor stage for each year from 1998 to 2019 (**Supplementary Figure 8**). The mean age at diagnosis for prostate cancer patients with different tumor stages remained constant over the 22-year study period. There were significant differences between the mean ages of patients with different tumor stages, the mean age of patients with localized prostate cancer was 3.10 (95% CI:2.54~3.65) years higher than the mean age of patients with regional prostate cancer. The mean age of patients with localized prostate cancer was 4.72 (95% CI:4.28~5.17) years lower than the mean age of patients with distant metastases; The mean age of patients with regional prostate cancer was 7.82 (95% CI:7.39~8.25) years lower than that of patients with distant metastases.

### 3.5 Survival

The median (95% CI) survival time (months) for all patients was 157.00 (156.63~157.37). For the different age groups, patients in the ≥80 age group had the shortest survival time with a median (95% CI) of 49.00 (48.63~49.37), while patients < 60 years had the longest survival time with a median (95% CI) of 304.00 (301.79~306.21). the median (95% CI) survival time for patients in the 60~69, 70~79 age groups were 202.00 (201.33~202.70) and 119.00 (118.59~119.42), respectively (**Supplementary Figure 9**).

Among prostate cancers with different tumor stages, patients with distant metastatic prostate cancer had the shortest survival time with a median (95% CI) of 26.00 (25.66~26.34). The median (95% CI) survival times for patients with localized and regional prostate cancer were 187.00 (186.44~187.56) and 217.00 (215.16~218.84) (**Supplementary Figure 10**). For different tumor grade, patients with G4 prostate cancer had the shortest survival time with a median (95% CI) of 48.00 (45.49~50.51). Patients with G2 prostate cancer had the longest survival time with a median (95% CI) of 185.00 (184.46~185.54). Patients with G1 and G3 prostate cancer had a median (95% CI) survival time with 140.00 (138.91~141.09) and 143.00 (142.40~143.60), respectively (**Supplementary Figure 11**). All these survival analyses were statistically significant (P<0.05).

We further evaluated 3-year, 6-year and 9-year survival patterns according to tumor stage and tumor grade. The 3-year, 6-year and 9-year survival rates for patients with localized and regional prostate cancer were mostly higher than 80%, and the survival rates were relatively high. The 9-year survival rates of patients with localized G1 and G3 prostate cancer were (77.75 ± 0.48%) and (71.12 ± 0.15%), The 6-year and 9-year survival rates of patients with localized G4 prostate cancer (66.21 ± 2.19%) and (50.76 ± 2.36%) were relatively low; The 9-year survival rates of patients with regional G3 prostate cancer were (77.14 ± 0.25%), the 3-year, 6-year and 9-year survival rates of patients with regional G4 prostate cancer (70.69 ± 3.29%), (57.87 ± 3.59%) and (50.23 ± 3.69%) were relatively low; In distant metastatic prostate cancer survival rates were low for all tumor grades, among which the survival rate of G4 prostate cancer is the worst, the 3-year, 6-year and 9-year survival rate were (19.44 ± 2.84%), (10.24 ± 2.21%) and (4.98 ± 1.64%), respectively (**Table 4**).

**Table 4:** Survival analysis of patients with prostate cancer: actuarial survival of prostate cancer patients by tumor stage and tumor grade.

Tumor grade	Localized				Regional				Distant			
	Median Survival (months)	Survival Rate (%)			Median Survival (months)	Survival Rate (%)			Median Survival (months)	Survival Rate (%)		
		3Year	6Year	9Year		3Year	6Year	9Year		3Year	6Year	9Year
Overall	187	94.05	85.86	76.45	217	94.79	87.78	80.08	26	40.97	21.00	12.78
G1	179	95.79	88.98	77.75	240	95.91	90.45	83.07	54	64.86	36.48	24.32
G2	201	95.38	88.52	80.12	245	97.31	92.85	87.18	48	63.47	40.26	28.69
G3	163	92.08	81.88	71.12	198	94.07	85.90	77.14	30	45.93	23.75	14.09
G4	104	82.98	66.21	50.76	94	70.69	57.87	50.23	17	19.44	10.24	4.98

Overall, survival rates for localized, regional and distant metastatic prostate cancer improved from 3-year survival rates in 1998 (91.50 ± 0.24%), (92.56 ± 0.49%) and (38.04 ± 1.51%) to 3-year survival rates in 2016 (95.34 ± 0.12%), (95.04 ± 0.27%) and (44.79 ± 0.85%); from 6-year survival rates in 1998 (80.40 ± 0.34%), (83.47 ± 0.70%) and (20.52 ± 1.26%) to 6-year survival rates in 2013 (87.90 ± 0.18%), (88.03 ± 0.45%) and (17.29 ± 0.73%), respectively; from 9-year survival rates in 1998 (68.97 ± 0.39%), (73.85 ± 0.83%) and (12.71 ± 1.04%) to 9-year survival rates (79.14 ± 0.21%), (80.89 ± 0.51%) and (11.49 ± 0.68%) in 2010, respectively (**Supplementary Figures 12**–**14**).

### 3.6 General clinical information and univariate and multivariate cox regression

General clinical information is shown in **Table 5**. median follow-up was 69 months and 4 261 cases of specific death. Univariate and multifactorial Cox regression analysis revealed that tumor stage, race, PSA, age and gleason score were independent risk factors for specific survival in patients with prostate cancer (P<0.05) (**Table 6**).

**Table 5:** General clinical data of training set and validation set samples of prostate cancer patients in SEER Database[*n*(%)].

Characteristic	All patients (*n*=105135) [n(%)]	Training set (*n*=70090) [n(%)]	Validation set (*n*=35045) [n(%)]	Χ^2^	*P*
Tumor grade				62.694	<0.001
G1	9722(9.2)	6424(9.2)	3298(9.4)		
G2	41757(39.7)	27302(39.0)	14455(41.2)		
G3	53567(51.0)	36300(51.7)	17267(49.3)		
G4	89(0.1)	64(0.1)	25(0.1)		
Tumor stage				25.590	<0.001
Localized	85669(81.5)	56816(81.1)	28853(82.3)		
Regional	16625(15.8)	11355(16.2)	5270(15.1)		
Distant	2841(2.7)	1919(2.7)	922(2.6)		
Race				1513.487	<0.001
White people	81188(77.2)	56424(80.5)	24764(70.7)		
Black people	16234(15.4)	8750(12.5)	7484(21.3)		
Others	7713(7.4)	4916(7.0)	2797(8.0)		
Bone metastasis				0.837	0.371
No	102465(97.5)	68288(97.4)	34177(97.5)		
Yes	2670(2.5)	1802(2.6)	868(2.5)		
Lung metastasis				1.349	0.276
No	105040(99.9)	70032(99.9)	35008(99.9)		
Yes	95(0.1)	58(0.1)	37(0.1)		
PSA(ng/ml)				21.975	<0.001
<4	13460(12.8)	9153(13.1)	4307(12.3)		
4.1~10	64215(61.1)	42566(60.7)	21649(61.8)		
10.1~20	16587(15.8)	11193(16.0)	5394(15.4)		
>20	10873(10.3)	7178(10.2)	3695(10.5)		
Age(Y)				151.174	<0.001
<60	26736(25.4)	17223(24.6)	9513(27.1)		
60~69	46760(44.5)	31019(44.3)	15741(44.9)		
70~79	25837(24.6)	17756(25.3)	8081(23.1)		
≥80	5802(5.5)	4092(5.8)	1710(4.9)		
Gleason score				273.069	<0.001
≤6	41530(39.5)	26486(37.8)	15044(42.9)		
7	43772(41.6)	30220(43.1)	13552(38.7)		
8~10	19833(18.9)	13384(19.1)	6449(18.4)		

**Table 6:** Univariate and multivariate analysis of prognostic factors related to specific survival in patients with prostate cancer.

Characteristic	Univariate analysis		Multivariate analysis	
	*HR* (95%*CI*)	*P*	*HR* (95%*CI*)	*P*
Tumor grade				
G1	Reference value			
G2	1.792(1.301~2.468)	<0.001		
G3	11.453(8.415~15.588)	<0.001		
G4	34.352(19.731~59.807)	<0.001		
Tumor stage				
Localized	Reference value		Reference value	
Regional	2.223(2.048~2.412)	<0.001	1.427(1.310~1.554)	<0.001
Distant	44.854(41.878~48.042)	<0.001	8.582(7.900~9.323)	<0.001
Race				
White people	Reference value		Reference value	
Black people	1.185(1.094~1.283)	<0.001	1.221(1.126~1.325)	<0.001
Others	0.948(0.840~1.069)	0.385	0.650(0.576~0.733)	<0.001
Bone metastasis				
No	Reference value			
Yes	36.943(34.618~39.425)	<0.001		
Lung metastasis				
No	Reference value			
Yes	23.791(17.997~31.451)	<0.001		
PSA(ng/ml)				
<4	Reference value		Reference value	
4.1~10	1.007(0.875~1.158)	0.926	0.868(0.754~0.999)	0.048
10.1~20	3.058(2.646~3.534)	<0.001	1.517(1.310~1.758)	<0.001
>20	14.555(12.727~16.645)	<0.001	2.579(2.233~2.977)	<0.001
Age(Y)				
<60	Reference value		Reference value	
60~69	1.214(1.104~1.334)	<0.001	1.067(0.970~1.173)	0.180
70~79	2.398(2.182~2.634)	<0.001	1.671(1.518~1.839)	<0.001
≥80	8.985(8.122~9.939)	<0.001	2.984(2.683~3.318)	<0.001
Gleason score				
≤6	Reference value		Reference value	
7	2.970(2.619~3.368)	<0.001	2.210(1.945~2.512)	<0.001
8~10	23.949(21.358~26.854)	<0.001	7.843(6.915~8.895)	<0.001

### 3.7 Development and validation of a nomogram model for patient-specific survival in prostate cancer

**Figure 6:**
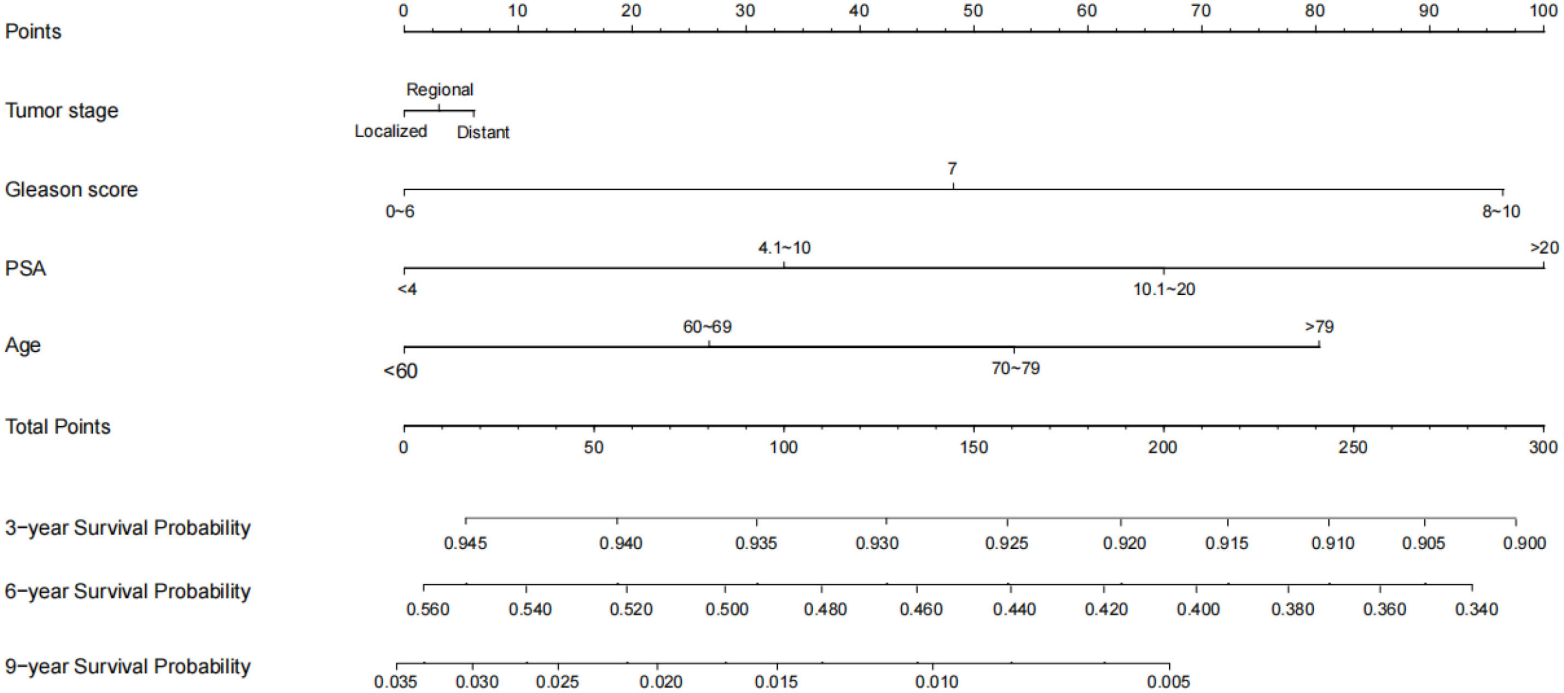
Nomogram of 3, 6, 9-year specific survival prediction of prostate cancer patients.

**Figure 7:**
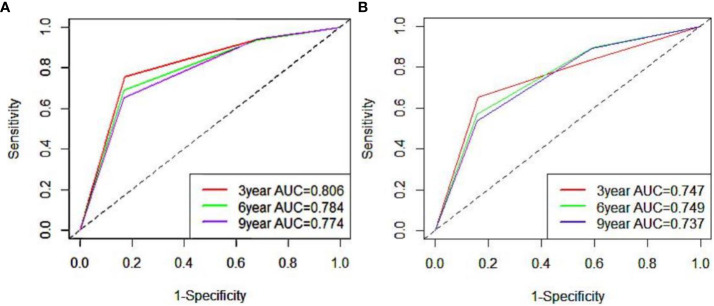
ROC curve of 3, 6 and 9 years of nomogram prediction model (**A**:training set; **B**:validation set).

**Figure 8:**
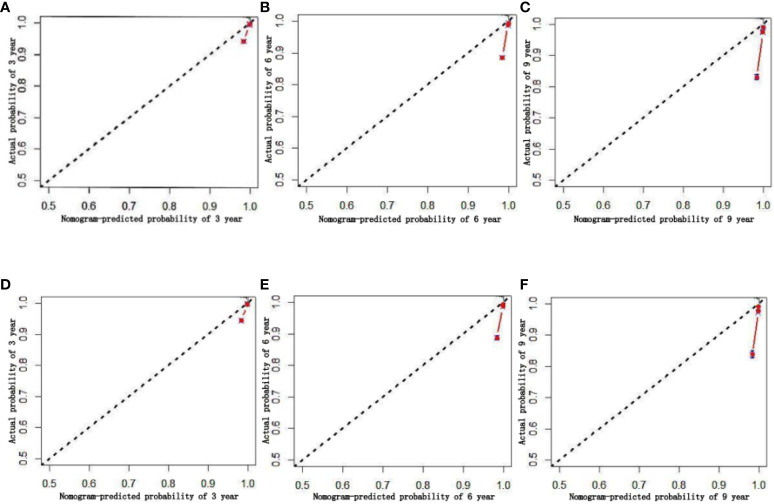
Calibration chart of 3、6、9-year specific survival probability (**A**–**C**:training set; **D**–**F**:validation set).

To ensure the accuracy of the model, the factors that had the greatest influence on the specific survival rate of prostate cancer patients were screened based on the AIC and C index. It was found that the model constructed based on four factors: tumor stage, PSA, age and gleason score, had the highest C-index (**Table 7**), indicating that the nomogram built based on the above four factors could accurately assess the specific survival rate of prostate cancer patients at 3, 6 and 9 years (**Figure 6**). Finally, four indicators/variables, including Tumor stage, Gleason score, PSA, and Age were retained in the regression equation. Cox regression model: h (t, x)=h0 (t) exp (1.049X_1_ + 1.064X2 + 0.540X_3_ + 0.453X_4_), with independent variables: X_1_ = Tumor stage, X_2_ = Gleason score, X_3_ = PSA, and X_4_ = Age (**Table 8**). The areas under the ROC curves were 0.806, 0.784, and 0. 774 for the training set at 3, 6, and 9 years, respectively. The areas under the ROC curves were 0.747, 0.749, and 0. 737 for the validation set at 3, 6, and 9 years, respectively (**Figure 7**), which had good reference value. The calibration curve was used for internal validation, with the X-axis representing the predicted mortality rate and the Y-axis representing the actual mortality rate. Both sets of data are seen to fit close to the diagonal line, indicating that the actual curve fits well with the ideal curve, and there was good agreement between the model-predicted overall survival rates and the true values at 3, 6, and 9 years (**Figure 8**).

**Table 7:** Consistency index of clinical factors in training set and validation set and *AIC* value of each factor.

Variable	*AIC*	Training set		Validation set	
		*C index*	95%*CI*	*C index*	95%*CI*
Gleason score	316515	0.789	0.760~0.818	0.752	0.709~0.795
PSA(ng/ml)	316401	0.693	0.654~0.732	0.696	0.645~0.747
Age(Y)	316366	0.641	0.602~0.680	0.632	0.581~0.683
Tumor stage	316342	0.736	0.701~0.771	0.738	0.691~0.785
Race	316342	0.513	0.484~0.542	0.520	0.479~0.561
Gleason score/PSA		0.820	0.791~0.849	0.801	0.769~0.851
Gleason score/PSA/Age		0.828	0.799~0.857	0.811	0.774~0.848
Gleason score/PSA/Age/Tumor stage		0.845	0.818~0.872	0.835	0.798~0.872
Gleason score/PSA/Age/Race		0.826	0.800~0.855	0.811	0.774~0.848
Gleason score/PSA/Age/Tumor stage/Race		0.844	0.817~0.871	0.834	0.795~0.873

**Table 8:** Variable evaluation table.

Variable	evaluation
X_1_=Tumor stage	0=Localized, 1=Regional, 2=Distant
X_2_=Gleason score	0 = 0~7, 1 = 7, 2 = 8~10
X_3_=PSA	0 = 0~4, 1 = 4.1~10, 2 = 10.1~20, 3= ≥20.1
X_4_=Age	0=<60, 1 = 60~69, 2 = 70~79, 3= ≥80

**Figure 9:**
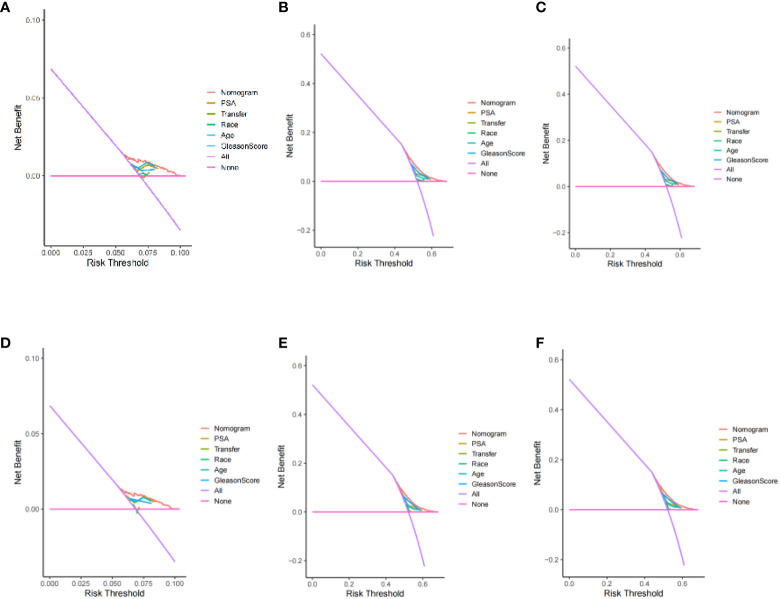
Decision curve analysis of 3、6、9-year specific survival probability (training set: **A**: 3 year; **B**: 6 years; **C**: 9 years) (validation set: **D**: 3 year; **E**: 6 years; **F**: 9 years).

After evaluating the accuracy of the model, reevaluate whether the inclusion of four factors can benefit prostate cancer patients in clinical practice. Using Decision Curve Analysis (DCA) to evaluate the net benefits of patients, calculate the clinical value of the model and its impact on actual decision-making. The Y-axis represents the calculated benefits, the X-axis represents the risk threshold, and the wavy line of the nomogram is further away from the intersection of the line, closer to the upper right, indicating greater clinical benefits. The results indicate that, the prediction model constructed based on four factors had more clinical benefits for patients compared to individual prediction models for each factor (**Figure 9**).

Application of nomogram: First, number each patient, then select any ID number to view the patient’s information and calculate the patient’s survival rate. For patient number 10035, Tumur stage=Regional, Gleason score=7, PSA=6.2ng/ml, Age=65 year. Its score is: 2.5 (Tumur stage=Regional)+47.5 (Gleason score=7)+32.5 (PSA=6.2ng/ml)+27.5 (Age=65 year)=110. The corresponding 3-year survival rate, 6-year survival rate, and 9-year survival rate of prostate cancer patients with a total score of 110 are 93.25%, 48.00%, and 1.30%.

## 4 Discussion

In this population-based study, we analyzed prostate cancer epidemiology and prognostic factors using data from a large number of prostate cancer patients reported in the SEER database from 1975 to 2019. The overall incidence of prostate cancer remained constant over 45 years, which is consistent with trends found in earlier epidemiological studies (9). We have analyzed many details of prostate cancer incidence trends and found a decrease in incidence trends across tumor stages, with the greatest decrease in localized prostate cancer, which may be due to the impact of effective preventive measures on prostate cancer incidence or the decreasing rate of patients undergoing PSA testing in the last decade or the prevalence of preventive measures for prostate cancer (10). Among the different tumor grade, except for G4 prostate cancer for which there has been a decrease, the incidence trend of prostate cancer has increased in all tumor grades, with the greatest increase in G2 prostate cancer.

Changes in patient management and disease related regulations during the period from 1975 to 2020 affect the incidence rate, prevalence, survival rate and other patient outcomes of prostate cancer. Several screening studies from the late 1980s to the early 1990s showed that, compared with the assessment of palpable tumors by digital rectal examination, PSA detection could identify more prostate cancer in the clinical local stage of organ limitation, especially in the United States, which led to a rapid rise in the incidence rate of prostate cancer (11–16).

This study shows that the incidence rate of prostate cancer increased sharply from 1988 to 1992, and reached the peak in incidence rate. This may be due to the extensive introduction of PSA monitoring (officially approved by FDA in 1986), which increased the detection of asymptomatic diseases. After that, the incidence rate of prostate cancer began to decline, which may be related to the recommendation of the United States Preventive Services Task Force (USPSTF) in 2008 to screen men ≥ 75 years old. Around 2012, the incidence rate of prostate cancer began to stabilize slowly, which may be due to concerns about over diagnosis and over treatment of prostate cancer. The U.S. Preventive Services Working Group recommended changing PSA to routine testing (16, 17). Therefore, after years of “excitement”, clinical doctors are starting to test fewer and fewer patients. It is worth noting that the change trend of incidence rate is parallel to the acceptance of PSA screening in some regions such as the United States, Europe and Australia. incidence rate is greatly affected by PSA testing and related screening plans (18). It can be considered that as long as there is screening, the incidence rate will increase.

In addition, these changes in patient management and disease-related regulations may also affect patient survival and other prognostic factors. In the early 1990s, the emergence of PSA screening also led to a shift in the diagnosis stage of prostate cancer, with an increase in the proportion of men diagnosed with localized diseases. Early detection and treatment of prostate cancer improved patient survival and other prognostic factors. Since 2008, the decrease in PSA testing has led to an increase in the number of late stage prostate cancer patients and a decrease in the number of early stage prostate cancer patients, which has led to poor treatment outcomes and a decrease in patient survival rates for most late stage patients.

In addition to PSA, medical imaging has always been a key component of early detection of prostate cancer (19). Medical imaging and other examination methods will also affect the incidence rate, prevalence, survival rate and other prognosis of prostate cancer. Hricak et al (20) published the first application of mpMRI in the prostate in 1983. Since then, mpMRI has been increasingly used for the diagnosis of prostate cancer (21). Many studies have confirmed the diagnostic reliability of mpMRI in detecting prostate cancer (22, 23). In the past, the lack of consistency in the diagnostic criteria of mpMRI led to differences in the number of patients diagnosed with prostate cancer in different regions, affecting the accuracy of the incidence rate of prostate cancer (24). In addition, different medical imaging equipment and quality applied in different regions will also affect the number of prostate cancer patients in different regions. For example, imaging examinations with high sensitivity may diagnose more patients, leading to an increase in the incidence rate of prostate cancer; Imaging examination with low sensitivity may diagnose a small number of patients, leading to a decline in the incidence rate of prostate cancer, and the incidence rate may be underestimated. In addition, the study found that mpMRI has less diagnosis of low-risk diseases and more diagnosis of high-risk diseases, which may lead to the underestimation of the incidence rate of low-risk prostate cancer and the overestimation of the incidence rate of high-risk prostate cancer (25). In order to standardize the evaluation of prostate imaging examination results, the European Society of Urogenital Radiology (eSUR) released an expert consensus based guideline in 2012: Prostate Imaging Report and Data System (PiraS). In 2015, the American College of Radiologists published a revised version. These guidelines provide clear diagnostic criteria for the Likert score of multi parameter series, and further correct the accuracy of the incidence rate of prostate cancer (26).

In addition, the improvement of mpMRI technology has generated more information about tumor characteristics, which may help improve surgical planning and patient prognosis. For example, mpMRI has good sensitivity in identifying multifocal, seminal vesicle invasion, and extracapsular dilation (27–30). The high sensitivity of imaging examination methods allows doctors to grasp the important disease conditions of patients and choose the best treatment method. In addition, mastering more disease information during the surgical process can ensure the accuracy of doctors’ surgical operations, avoid various medical accidents caused by unfamiliarity with the condition, thereby improving patient survival rate, improving patient prognosis, and prolonging patient life.

Imaging examination methods can not only affect the diagnosis of prostate cancer, but also affect the positive rate of surgical margins. The positive rate of surgical margins affects patients’ later tumor recurrence and metastasis, thereby affecting their prognosis and survival time. Research has shown that there is a statistically significant correlation between the probability of receiving MPMRI before surgery and the lower probability of positive surgical margins (31). Cole et al^[31^ found that the mpMRI group had a lower probability of positive surgical margins, their propensity score weighted sensitivity analysis also found that the probability of surgical margin positivity was lower in males who underwent MRI examination. Another report from Stockholm stated that the positive margin rate in the mpMRI group (26.7%) was significantly lower than that in the non MRI group (33%) (32). Cole et al (31) found that the proportion of men who underwent MRI examination before surgery increased from 2.9% in 2004 to 28.2% in 2015. An increase in the proportion of men who underwent MRI examination before surgery may reduce the positive rate of surgical resection, improve the patient’s condition and prognosis, and prolong their lifespan. The contents discussed above may lead to certain inevitable differences in the incidence rate, prevalence and survival rate of prostate cancer. However, as the most authoritative and representative database in the United States, SEER database can represent the epidemiological characteristics of local prostate cancer in this study.

Because our study found that the incidence and prevalence of prostate cancer remain at a high level, and more relevant studies are needed to evaluate the best treatment for these patients. So in this study, we performed a survival analysis using the SEER database and confirmed the significance of early diagnosis of age, tumor stage and tumor grade in prognosis. Our findings are consistent with other studies in that patients over 80 years of age had a poor prognosis, whereas patients under 60 years of age had the best prognosis; patients with G4 prostate cancer had a poor prognosis, whereas patients with G2 prostate cancer had the best prognosis. In our analysis, patients with localized and regional prostate cancer at the time of diagnosis had a better prognosis than patients with distant metastatic prostate cancer. This result highlights the importance of early detection and treatment of prostate cancer. This is consistent with the results of Gandaglia’s (33) studies. For the entire cohort, survival rates improved over time, and this improvement may be related to advances in anticancer therapy, including the availability and use of targeted therapies.

Currently, the main evaluation index for prostate cancer survival is TNM staging, but TNM staging is not able to accurately assess patient survival (6), and more reliable evaluation indexes or prediction tools need to be explored, and nomograms are currently more commonly used tools for cancer prognosis evaluation, which can more accurately estimate the probability of a specific event for each individual by incorporating multiple risk factors compared to a single evaluation index (34, 35). However, there is no prognostic prediction model for prostate cancer constructed based on large sample data. Therefore, in this study, we screened prostate cancer prognostic influencing factors and built a prognostic model based on the information of prostate cancer patients in the SEER database to provide a reference basis for the assessment of patient prognosis.

We performed a multivariate survival analysis using a Cox regression. We found that age, race, tumor stage, PSA, and gleason score were associated with patient-specific survival in prostate cancer patients. Further analysis revealed that gleason score, tumor stage, age and PSA had the greatest impact on patient-specific survival in prostate cancer. gleason score is the main indicator for treatment selection and assessment of prognosis, and as gleason score increases, patient survival decreases along with gleason score (36). Previous studies have indicated that patients with prostate cancer with a pathological gleason score ≥8 have a high rate of positive seminal vesicle invasion、 cut margins、earlier biochemical recurrence、shorter survival time, therefore should be more aware of prognostic monitoring and follow-up (37). The results of this study suggest that gleason score is an important influential factor in the prognosis of prostate cancer patients, and patients with high gleason score have a lower specific survival rate. This is consistent with the findings of Ohtaka (38). Studies have shown that tumor metastasis can cause deterioration in the function of other tissues and organs, in addition, embolization of tumors in blood vessels may even cause vascular embolism, all of which can lead to poor physical condition and shortened survival. The results of this study showed that patients with metastatic prostate cancer had a lower survival rate. This is consistent with the results of studies by Hao (39) and DeSantis (40).

Age is closely related to the prognosis of prostate cancer, and the results of this study showed that the older the age, the worse the prognosis of the patients, with the best prognosis in patients <60 years old. This is consistent with the findings of Matsushita (41). Therefore, knowledge about urological and prostate cancer-related diseases and regular physical examinations are needed for prevention or early detection of urological-related diseases in the higher age groups. Previous studies have shown that PSA is an independent risk factor for the prognosis of prostate cancer patients, and high PSA levels are associated with a high risk of cancer death. However, recent studies found no relationship between PSA levels and prognosis in mPCa patients (42). the effect of PSA on the prognosis of prostate cancer is still controversial. Therefore, PSA must be combined with other factors when determining prognosis. The results of this study showed that patients with PSA (4.1-10 ng/ml) had a better prognosis than other groups of patients. This is consistent with the results of the study by Zijian Tian (43).

With the results of survival analysis, our nomogram including 4 important prognostic parameters (age, PSA, tumor stage and gleason score) can provide simple and accurate prognostic prediction for prostate cancer patients. For example, according to our nomogram, a patient with prostate cancer aged 65 years (26 points), PSA 13.0 ng/ml (67 points), gleason score 7 (48 points) and regional (3 points) had a 3-year survival rate of 92.7% (144 points), a 6-year survival rate of 45.0% (144 points) and a 9-year survival rate of 0.9% (144 points). Overall, this simple and effective tool can more accurately evaluate the survival of patients through various parameters of prostate cancer patients, thus facilitating clinical decision making and communication with patients and their families.

### 4.1 Limitations and advantages

Our study has several limitations. First, the SEER database may not capture all prostate cancer cases; therefore, we may actually underestimate the true incidence and prevalence of prostate cancer. Although the SEER database has a large sample size, it lacks important treatment information such as perioperative chemotherapy and postoperative chemotherapy, and the database includes patients over a large time span, and with the gradual development of medical technology, treatment varies from period to period, and this study is not yet able to correct for these possible confounding factors. In addition, novel targeted therapies have been used to treat patients with localized advanced or distant metastases with good survival benefits in selected cohorts over the past decades, and this information may confound the results of the survival analysis and may lead to differences in survival benefits in patients from different time periods. In addition, after establishing a nomogram model in this study, we only conducted internal validation using our data from this database to verify the accuracy of the model. We found that the model had good predictive accuracy, but the internal validation was not convincing and required external validation using other datasets. However, due to the inability to find suitable data other than the database, external validation was not conducted. Therefore, in the later stage, we need to find a suitable dataset for external validation. In addition, our hospital has also started collecting relevant data for further external validation to improve the prediction accuracy of the model and expand its application scope.

However, our research also has several advantages. To our knowledge, this study is one of the largest and latest explorations in cancer, the SEER database used in this study is the most authoritative and representative database in the United States, its data size and long-term follow-up data largely compensate for the shortcomings, and provide comprehensive epidemiological and survival data related to cancer, which can represent the epidemiological characteristics of local prostate cancer.

## 5 Conclusion

In this study, the incidence of prostate cancer remained unchanged over 45 years, but the incidence of prostate cancer with different characteristics changed significantly. In terms of survival, there were differences in survival rates by tumor stage and tumor grade. However, outcomes generally improved with advances in diagnosis and treatment. In addition, a new nomogram was established and validated in this study that can effectively predict 3-, 6-, and 9-year survival rates in prostate cancer patients. It can provide accurate and useful information for physicians and patients and guide treatment strategies for prostate cancer patients.

## Data availability statement

Publicly available datasets were analyzed in this study. This data can be found here: Surveillance, Epidemiology, and End Results Program (SEER) database.

## Author contributions

Conception and design: SA, NT, and HN. Collection and assembly of data: SA and KB. Data analysis and interpretation: SA, YM, and AM. Manuscript writing: All authors. All authors contributed to the article and approved the submitted version.
