# Supplementary material for: Prostate cancer epidemiology and prognostic factors in the United States
Source: Front Oncol. 2023 Oct 12;13:1142976. doi: 10.3389/fonc.2023.1142976 (PMC10603232; doi:10.3389/fonc.2023.1142976)
Supplement: Supplementary file 1 [file Table_1.docx]

Supplementary Table 1 Annual percent change (APC) and average APC (AAPC) in rates per 100 000 men of prostate cancer, by selected characteristics - U.S. Cancer Statistics, United States, 1975-2019

| Characteristic | APCC | |  | APC | | | | | | | | | | | |
| --- | --- | --- | --- | --- | --- | --- | --- | --- | --- | --- | --- | --- | --- | --- | --- |
|  | Year | AAPC(95%CI) |  | Year | APC1(95%CI) | Year | APC2(95%CI) | Year | APC3(95%CI) | Year | APC4(95%CI) | Year | APC5(95%CI) | Year | APC6(95%CI) |
| Overall | 1975-2019 | 0.45  (-0.87~1.80) |  | 1975-1986 | 1.50  (-0.52~3.55) | 1986-1992 | 11.36  (6.26~16.71) | 1992-1995 | -7.55  (-20.49~7.48) | 1995-2009 | -0.68  (-1.28~-0.07) | 2009-2014 | -7.96  (-10.75~-5.08) | 2014-2019 | 2.75  (0.58~4.98) |
| Age  gruop(y) |  |  |  |  |  |  |  |  |  |  |  |  |  |  |  |
| 15-54 | 1975-2019 | 4.03  (2.73~5.34) |  | 1975-1985 | -0.50  (-5.07~4.28) | 1985-1999 | 14.64  (12.70~16.64) | 1999-2009 | 3.54  (2.60~4.49) | 2009-2014 | -7.46  (-10.34~-4.50) | 2014-2019 | -1.71  (-4.16~0.80) |  |  |
| 55-64 | 1975-2019 | 2.50  (0.96~4.05) |  | 1975-1988 | 3.20  (1.05~5.40) | 1988-1992 | 23.25  (6.24~42.98) | 1992-2009 | 0.62  (0.06~1.18) | 2009-2014 | -7.81  (-10.73~-4.80) | 2014-2019 | 2.86  (0.55~5.23) |  |  |
| 65-74 | 1975-2019 | 1.21  (-0.11~2.54) |  | 1975-1986 | 2.45  (0.46~4.47) | 1986-1992 | 14.75  (9.71~20.02) | 1992-1995 | -6.95  (-19.35~7.36) | 1995-2010 | -0.84  (-1.40~-0.29) | 2010-2014 | -9.25  (-13.64~-4.64) | 2014-2019 | 3.40  (1.24~5.61) |
| 75-84 | 1975-2019 | -0.79  (-1.95~0.38) |  | 1975-1986 | 1.33  (-0.33~3.03) | 1986-1992 | 9.26  (5.24~13.44) | 1992-1995 | -14.84  (-25.70~-2.39) | 1995-2007 | -1.63  (-2.41~-0.85) | 2007-2014 | -7.76  (-9.37~-6.13) | 2014-2019 | 4.48  (1.96~7.07) |
| ≥85 | 1975-2019 | -2.50  (-3.43~1.57) |  | 1975-1988 | 0.52  (-0.24~1.28) | 1988-1991 | 12.39  (-0.20~26.56) | 1991-1995 | -13.86  (-17.93~-9.58) | 1995-2004 | -2.54  (-3.47~-1.60) | 2004-2012 | -7.31  (-8.23~-6.38) | 2012-2019 | -1.39  (-2.40~-0.37) |
| White people | 1975-2019 | 0.31  (-1.01~1.65) |  | 1975-1986 | 1.71  (-0.26~3.72) | 1986-1992 | 11.47  (6.48~16.70) | 1992-1995 | -8.65  (-21.55~6.38) | 1995-2008 | -0.52  (-1.25~0.23) | 2008-2014 | -7.26  (-9.35~-5.13) | 2014-2019 | 1.84  (-0.47~4.20) |
| 15-54 | 1975-2019 | 3.61  (1.92~5.32) |  | 1975-1985 | -0.10  (-5.82~5.97) | 1985-1999 | 14.04  (11.52~16.61) | 1999-2009 | 3.19  (1.94~4.45) | 2009-2013 | -9.52  (-15.78~-2.78) | 2013-2019 | -3.01  (-5.38~-0.59) |  |  |
| 55-64 | 1975-2019 | 2.24  (0.96~3.53) |  | 1975-1987 | 2.89  (0.55~5.28) | 1987-1992 | 19.64  (9.29~30.96) | 1992-2009 | 0.47  (-0.08~1.01) | 2009-2014 | -8.62  (-11.53~-5.62) | 2014-2019 | 2.19  (-0.23~4.66) |  |  |
| 65-74 | 1975-2019 | 1.08  (-0.22~2.40) |  | 1975-1986 | 2.74  (0.78~4.74) | 1986-1992 | 14.84  (9.97~19.92) | 1992-1995 | -7.58  (-19.85~6.56) | 1995-2010 | -1.06  (-162~-0.50) | 2010-2014 | -9.56  (-14.03~-4.86) | 2014-2019 | 2.89  (0.65~5.18) |
| 75-84 | 1975-2019 | -0.89  (-2.06~0.29) |  | 1975-1986 | 1.52  (-0.03~3.10) | 1986-1992 | 8.89  (4.95~12.98) | 1992-1995 | -15.51  (-26.49~-2.88) | 1995-2007 | -1.67  (-2.46~-0.87) | 2007-2014 | -7.89  (-9.65~-6.08) | 2014-2019 | 4.31  (1.66~7.03) |
| 85- | 1975-2019 | -2.54  (-3.56~1.50) |  | 1975-1988 | 0.53  (-0.29~1.35) | 1988-1991 | 11.92  (-1.71~27.45) | 1991-1995 | -14.76  (-19.31~-9.96) | 1995-2003 | -1.96  (-3.25~-0.66) | 2003-2012 | -6.90  (-7.75~-6.04) | 2012-2019 | -1.39  (-2.54~-0.22) |
| Black people | 1975-2019 | 0.61  (-1.10~2.35) |  | 1975-1989 | 1.87  (0.26~3.51) | 1989-1992 | 18.05  (-7.36~50.43) | 1992-2010 | -1.45  (-1.82~-1.08) | 2010-2014 | -7.85  (-11.36~-4.20) | 2014-2019 | 2.02  (0.35~3.72) |  |  |
| 15-54 | 1975-2019 | 4.73  (2.20~7.31) |  | 1975-1989 | 0.28  (-3.75~4.48) | 1989-1994 | 32.38  (12.48~55.80) | 1994-1999 | 11.63  (3.54~20.35) | 1999-2010 | 3.48  (2.56~4.41) | 2010-2013 | -9.51  (-18.70~0.73) | 2013-2019 | -0.62  (-2.55~1.35) |
| 55-64 | 1975-2019 | 1.97  (-0.76~4.48) |  | 1975-1989 | 0.95  (-1.44~3.40) | 1989-1992 | 37.30  (-7.32~103.39) | 1992-2009 | -0.13  (-0.69~0.44) | 2009-2014 | -6.11  (-8.91~-3.22) | 2014-2019 | 2.28  (0.21~4.39) |  |  |
| 65-74 | 1975-2019 | 1.00  (-0.84~2.87) |  | 1975-1989 | 2.34  (0.68~4.04) | 1989-1992 | 20.85  (-6.74~56.59) | 1992-2010 | -1.57  (-2.00~-1.13) | 2010-2014 | -7.11  (-11.45~-2.57) | 2014-2019 | 2.52  (0.53~-4.55) |  |  |
| 75-84 | 1975-2019 | -0.97  (-3.19~1.31) |  | 1975-1989 | 1.77  (0.02~3.56) | 1989-1992 | 15.82  (-11.33~51.27) | 1992-1995 | -10.82  (-25.94~7.38) | 1995-2009 | -2.72  (-3.49~-1.93) | 2009-2014 | -9.83  (-13.51~-6.00) | 2014-2019 | 2.70  (-0.28~5.77) |
| 85- | 1975-2019 | -2.46  (-3.30~-1.62) |  | 1975-1991 | 2.18  (0.15~4.24) | 1991-2004 | -3.72  (-5.04~-2.37) | 2004-2019 | -6.13  (-6.98~-5.28) |  |  |  |  |  |  |
| AI/AN | 1992-2019 | -3.40  (-3.97~-2.82) |  | 1992-2019 | -3.40  (-3.97~-2.82) |  |  |  |  |  |  |  |  |  |  |
| 15-54 | 1992-2019 | -0.87  (-2.45~0.74) |  | 1992-2019 | -0.87  (-2.45~0.74) |  |  |  |  |  |  |  |  |  |  |
| 55-64 | 1992-2019 | -2.92  (-3.83~-2.00) |  | 1992-2019 | -2.92  (-3.83~-2.00) |  |  |  |  |  |  |  |  |  |  |
| 65-74 | 1992-2019 | -3.31  (-4.03~-2.59) |  | 1992-2019 | -3.31  (-4.03~-2.59) |  |  |  |  |  |  |  |  |  |  |
| 75-84 | 1992-2019 | -3.57  (-4.53~-2.61) |  | 1992-2019 | -3.57  (-4.53~-2.61) |  |  |  |  |  |  |  |  |  |  |
| 85- | 1992-2019 | -5.05  (-6.77~-3.29) |  | 1992-2019 | -5.05  (-6.77~-3.29) |  |  |  |  |  |  |  |  |  |  |
| AP | 1992-2019 | -2.74  (-4.14~-1.32) |  | 1992-1996 | -7.13  (-12.02~-1.96) | 1996-2001 | 2.24  (-2.42~7.11) | 2001-2010 | -3.08  (-4.31~-1.83) | 2010-2014 | -9.46  (-14.55~-4.06) | 2014-2019 | 2.33  (-0.18~4.90) |  |  |
| 15-54 | 1992-2019 | 1.61  (0.04~3.21) |  | 1992-2007 | 6.91  (4.31~9.57) | 2007-2019 | -4.63  (-6.59~-2.63) |  |  |  |  |  |  |  |  |
| 55-64 | 1992-2019 | 0.66  (-0.71~2.05) |  | 1992-2001 | 4.81  (2.49~7.18) | 2001-2010 | -0.50  (-2.10~1.13) | 2010-2014 | -8.63  (-14.64~-2.19) | 2014-2019 | 3.28  (0.18~6.47) |  |  |  |  |
| 65-74 | 1992-2019 | -1.99  (-3.34~-0.62) |  | 1992-2010 | -1.34  (-1.98~-0.70) | 2010-2014 | -10.24  (-17.50~-2.35) | 2014-2019 | 2.71  (-0.87~6.43) |  |  |  |  |  |  |
| 75-84 | 1992-2019 | -4.05  (-6.08~-1.98) |  | 1992-1998 | -7.51  (-9.92~-5.03) | 1998-2001 | 5.45  (-7.95~20.79) | 2001-2010 | -5.06  (-6.34~-3.78) | 2010-2013 | -14.97  (-26.02~-2.28) | 2013-2019 | 2.48  (0.15~4.86) |  |  |
| 85- | 1992-2019 | -6.03  (-6.58~-5.49) |  | 1992-2019 | -6.03  (-6.58~-5.49) |  |  |  |  |  |  |  |  |  |  |
| Disease Stage |  |  |  |  |  |  |  |  |  |  |  |  |  |  |  |
| Localized | 1998-2019 | -1.83  (-2.76~-0.90) |  | 1998-2001 | 5.98  (3.02~9.03) | 2001-2004 | -4.48  (-8.44~-0.34) | 2004-2007 | 1.77  (-2.45~6.18) | 2007-2011 | -5.25  (-7.21~-3.25) | 2011-2014 | -11.32  (-15.29~-7.16) | 2014-2019 | 1.99  (0.96~3.02) |
| Regional | 1998-2019 | -1.77  (-3.91~0.43) |  | 1998-2003 | -6.21  (-9.80~-2.48) | 2003-2010 | -0.04  (-2.47~2.45) | 2010-2013 | -7.92  (-20.19~6.24) | 2013-2019 | 3.34  (0.94~5.78) |  |  |  |  |
| Distant | 1998-2019 | 0.57  (-0.80~1.96) |  | 1998-2000 | -9.09  (-21.47~5.25) | 2000-2010 | -1.35  (-2.27~-0.42) | 2010-2019 | 5.08  (4.29~5.88) |  |  |  |  |  |  |
| Tumor Grade |  |  |  |  |  |  |  |  |  |  |  |  |  |  |  |
| G1 | 1975-2017 | 0.47  (-1.95~2.95) |  | 1975-1993 | 2.18  (0.95~3.43) | 1993-2011 | -21.64  (-23.06~-20.19) | 2011-2014 | 292.37  (184.47~441.21) | 2014-2017 | 3.22  (-3.76~10.71) |  |  |  |  |
| G2 | 1975-2017 | 2.99  (1.47~4.54) |  | 1975-1992 | 12.39  (11.14~13.66) | 1992-2002 | 2.12  (0.72~3.54) | 2002-2005 | -16.57  (-26.23~-5.64) | 2005-2008 | 0.01  (-12.07~13.74) | 2008-2013 | -10.74  (-14.74~-6.55) | 2013-2017 | 3.93  (-0.85~8.95) |
| G3 | 1975-2017 | 1.77  (0.08~3.48) |  | 1975-1992 | 6.37  (4.53~8.25) | 1992-2001 | -1.46  (-4.22~1.38) | 2001-2006 | 19.67  (14.32~25.27) | 2006-2012 | -3.35  (-5.62~-1.03) | 2012-2015 | -30.37  (-40.17~-18.95) | 2015-2017 | 11.08  (-5.73~30.87) |
| G4 | 1975-2017 | -10.39  (-13.86~-6.77) |  | 1975-1977 | -30.74  (-63.39~5.88) | 1977-1982 | 12.53  (-6.98~36.13) | 1982-2012 | -7.12  (-7.90~-6.34) | 2012-2017 | -33.38  (-44.80~-19.60) |  |  |  |  |
