# Supplementary material for: Prostate cancer epidemiology and prognostic factors in the United States
Source: Front Oncol. 2023 Oct 12;13:1142976. doi: 10.3389/fonc.2023.1142976 (PMC10603232; doi:10.3389/fonc.2023.1142976)
Supplement: Supplementary file 2 [file DataSheet_1.docx]

Supplementary Figures Legend

Supplementary Figure 1 Incidence of Prostate Cancer Over Time by Age in White People

Supplementary Figure 2 Incidence of Prostate Cancer Over Time by Age in Black People

Supplementary Figure 3 Incidence of Prostate Cancer Over Time by Age in American Indian and Alaska Native

Supplementary Figure 4 Incidence of Prostate Cancer Over Time by Age in Asian and Pacific Islander

Supplementary Figure 5 20-Year Limited Duration Prevalence of Prostate Cancer Over Time

Supplementary Figure 6 20-Year Limited Duration Prevalence of Prostate Cancer Over Time by Tumor Stage

Supplementary Figure 7 20-Year Limited Duration Prevalence of Prostate Cancer Over Time by Tumor Grade

Supplementary Figure 8 Trends in Mean Age at Diagnosis by Tumor Stage

Supplementary Figure 9 Survival Curve of Patients with Prostate Cancer by Age

Supplementary Figure 10 Survival Curve of Patients with Prostate Cancer by Tumor Stage

Supplementary Figure 11 Survival Curve of Patients with Prostate Cancer by Tumor Grade

Supplementary Figure 12 Trends in 3-Year Overall Survival Probabilities by Tumor Stage

Supplementary Figure 13 Trends in 6-Year Overall Survival Probabilities by Tumor Stage

Supplementary Figure 14 Trends in 9-Year Overall Survival Probabilities by Tumor Stage
